# Supplementary material for: Polyketide synthase gene clusters in the ericoid mycorrhizal fungus Oidiodendron maius: genome-wide prediction, expression profiling, and functional characterization of knockout mutants
Source: Front Plant Sci. 2026 Sep 16;17:1924430. doi: 10.3389/fpls.2026.1924430 (PMC13623903; doi:10.3389/fpls.2026.1924430)
Supplement: Supplementary file 1 [file SupplementaryFile1.zip › Supplementary Materials/Supplementary Folder 1.zip!/Oidma1_AssemblyScaffolds_Repeatmasked/knownclusterblast/cluster55/jgi.p_Oidma1_36233_mibig_hits.html]

| MIBiG Protein | Description | MIBiG Cluster | MiBiG Product | % ID | % Coverage | BLAST Score | E-value |
| --- | --- | --- | --- | --- | --- | --- | --- |
| AGO86660.1 | putative\_fungal\_transcription\_factor | BGC0001255 | Equisetin\_biosynthetic\_gene\_cluster | 34 | 96.2441314554 | 321 | 1.8e-87 |
| AGN71612.1 | putative\_transcriptional\_regulatory\_protein | BGC0000027 | Azaphilone\_biosynthetic\_gene\_cluster | 39 | 61.5023474178 | 280 | 3.6e-75 |
